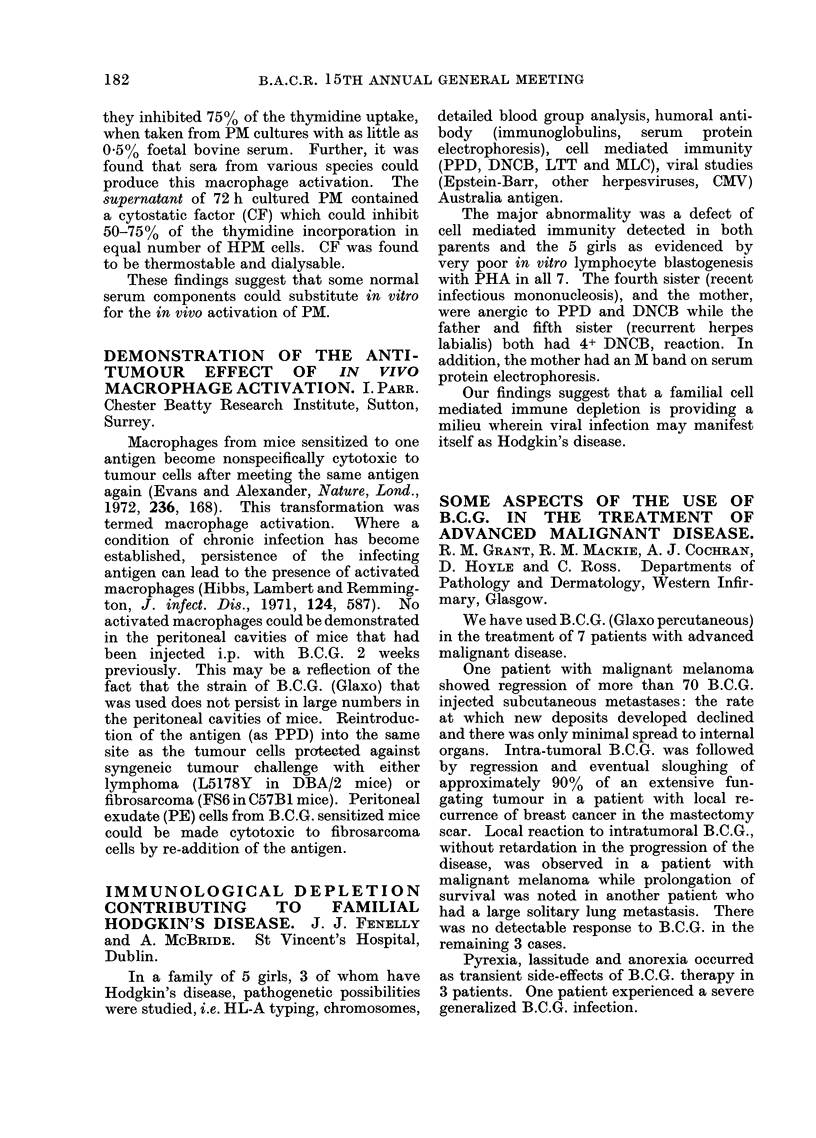# Proceedings: Immunological depletion contributing to familial Hodgkin's disease.

**DOI:** 10.1038/bjc.1974.163

**Published:** 1974-08

**Authors:** J. J. Fenelly, A. McBride


### IMMUNOLOGICAL DEPLETION CONTRIBUTING TO FAMILIAL HODGKIN'S DISEASE.

J. J. FENELLY and A. MCBRIDE. St Vincent's Hospital, Dublin.

In a family of 5 girls, 3 of whom have Hodgkin's disease, pathogenetic possibilities were studied, *i.e.* HL-A typing, chromosomes, detailed blood group analysis, humoral antibody (immunoglobulins, serum protein electrophoresis), cell mediated immunity (PPD, DNCB, LTT and MLC), viral studies (Epstein-Barr, other herpesviruses, CMV) Australia antigen.

The major abnormality was a defect of cell mediated immunity detected in both parents and the 5 girls as evidenced by very poor *in vitro* lymphocyte blastogenesis with PHA in all 7. The fourth sister (recent infectious mononucleosis), and the mother, were anergic to PPD and DNCB while the father and fifth sister (recurrent herpes labialis) both had $4^+$ DNCB, reaction. In addition, the mother had an M band on serum protein electrophoresis.

Our findings suggest that a familial cell mediated immune depletion is providing a milieu wherein viral infection may manifest itself as Hodgkin's disease.